# Solitary Extramedullary Plasmacytoma of the Left Lower Eyelid: A Case Report and Review of Literature

**DOI:** 10.7759/cureus.52718

**Published:** 2024-01-22

**Authors:** Ogochukwu Ugochukwu, Chukwunonso Ezeani, Marshall Stagg, Gregory Henkelmann

**Affiliations:** 1 Research and Development, Our Lady of the Lake Regional Medical Center, Baton Rouge, USA; 2 Internal Medicine Residency Program, Baton Rouge General Medical Center, Baton Rouge, USA; 3 Oncology, Our Lady of the Lake Regional Medical Center, Baton Rouge, USA; 4 Radiation Oncology, Our Lady of the Lake Regional Medical Center, Baton Rouge, USA

**Keywords:** extramedullary plasmacytoma (emp), plasma cell myeloma, plasma cell tumor, eyelid mass, solitary extramedullary plasmacytoma

## Abstract

Solitary extramedullary plasmacytoma (SEP) is a collection of plasma cells in soft tissue tumors characterized by monoclonal plasma cells without systemic symptoms or evidence of bone disease. We present a case of SEP in a 49-year-old African American patient who presented with a slowly enlarging eyelid mass and underwent an excisional biopsy with ophthalmology before the diagnosis was confirmed by pathology in the absence of systemic symptoms or bone disease. Our review found only six confirmed cases of SEP of the eyelid described in the literature. In such cases, treatment is typically surgical excision or radiotherapy. Our patient was treated with radiation after the excision was incomplete. This case report adds another rare case of SEP of the eyelid to the literature.

## Introduction

Plasma cell neoplasia or plasmacytomas are a group of disorders characterized by the neoplastic proliferation of a single clone of plasma cells, typically producing a monoclonal immunoglobulin. Plasma cell neoplasms can present as a single lesion (solitary plasmacytoma (SP)) or multiple lesions (multiple myelomas (MM)). Solitary plasmacytoma most frequently occurs in bone (plasmacytoma of bone) but can also be found in soft tissue (solitary extramedullary plasmacytoma (SEP)) [1].

Plasmacytomas may be primary or secondary tumors based on the presence of systemic disease, or MM. The malignant plasma cell neoplasm, or MM, may initially manifest as SP. Solitary extramedullary plasmacytomas are uncommon plasma cell tumors that develop in soft tissue as isolated tumors without osseous involvement. According to a comparative study in 2021, the incidence rates for SP, extramedullary plasmacytoma, and MM were 0.45, 0.09, and 8.47, respectively, per 100,000 persons between the years 2003 and 2016 [2]. Solitary extramedullary plasmacytoma has an incidence of 0.1 per 100,000 persons per year and typically affects patients between the ages of 50 and 80, with a median age of 55 to 60 years [1]. Solitary extramedullary plasmacytoma is seen mainly in the upper aerodigestive tract, accounting for 3% of plasma cell malignancies [3, 4]. Other sites include the gastrointestinal tract, bladder, breasts, thyroid, and lymph nodes [5]. Solitary extramedullary plasmacytoma rarely manifests around the eye; we have only found six confirmed cases of eyelid SEP described in the literature. Histopathologic examination of biopsy specimens and systemic evaluation to rule out MM establish the diagnosis. We report a primary extramedullary plasmacytoma of the left lower eyelid, presenting as a painless mass.

## Case presentation

A 49-year-old African-American female presented to our hospital complaining of a painless mass on the medial aspect of her left lower eyelid. The mass had been increasing in size over the last six months. She noted occasional blurred vision in the left eye but no other symptoms. Her medical history was significant for long-standing low back pain and lazy eye following childhood trauma. As a result, she experienced reduced vision in the left eye almost all her life. She also smoked half a pack of cigarettes daily and had a 12.5-pack-year smoking history.

She was afebrile (98.6F) on physical examination with a blood pressure of 155/79 mmHg. Other vital signs were stable. A 5 x 4 mm non-tender, non-erythematous mass was present on the medial canthus of the left lower eyelid. This mass was cystic on illumination and had a mucoid reflex plugging punctum. She also had dysconjugate gaze and poor vision in the left eye (this problem had been present since childhood). Visual acuity was 20/20 and 20/40 in the right and left eyes, respectively. The visual fields were full bilaterally. Extraocular movement and fundoscopy were normal. The rest of the physical examination was normal.

**Figure 1:**
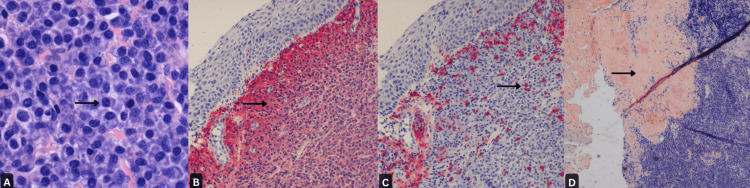
A: Collection of plasma cells, positive for CD 138 (x400); B: Plasma cells showing kappa light chain restriction by in-situ hybridization (100x); C: Lambda light chain in-situ hybridization highlights only rare, scattered plasma cells (x100); D: Congo red stain showing orangeophilic material consistent with amyloid deposition (x40).

An excisional biopsy of the mass with lacrimal probing followed by immunohistochemical stains showed monoclonal malignant plasma cells with kappa chain restriction consistent with plasma cell neoplasm (Figures 1A-1D).

Multiple myeloma workups were unremarkable, including serum protein electrophoresis, immunoglobulin levels, and serum free light chains. She tested positive for hepatitis C and was treated with sofosbuvir/velpatasvir (Table 1).

**Table 1:** Laboratory parameters on presentation

Laboratory parameter	Value	Reference levels
Hemoglobin	15.3	12.0-16.0 g/dL
White blood cell	6.8	4.0-11.0 1000/uL
Creatinine	0.82	0.57-1.25 mg/dL
Calcium	10.5	8.8-10.6 mg/dL
Total protein	8.2	6.0-8.3 g/dL
Albumin	4.2	3.5-5.0 g/dL
Hepatitis C viral load	4,940,000	IU/mL
Immunoglobulin M	46.3	52-318 mg/dL
Immunoglobulin G	1,167	540-1,877 mg/dL
Immunoglobulin A	183.9	38-397 mg/dL
Albumin densitometer	4.38	3.50-5.0 gm/dl
Alpha 1	0.32	0.10-0.40 gm/dl
Alpha 2	0.96	0.20-0.90 gm/dl
Beta	1.28	0.50-1.30 gm/dl
Gamma	1.26	0.70-1.80 gm/dl
Free kappa light chains	22.5	3.3-19.4 mg/L
Free lambda light chains	11.4	5.7-26.3 mg/L
Kappa/lambda ratio	1.97	0.26-1.65

A positron emission tomography (PET) scan showed no suspicious bone lesions and a bone marrow biopsy showed normal cells with less than 1% plasma cells and no blasts, confirming the diagnosis of extramedullary plasmacytoma.

**Figure 2:**
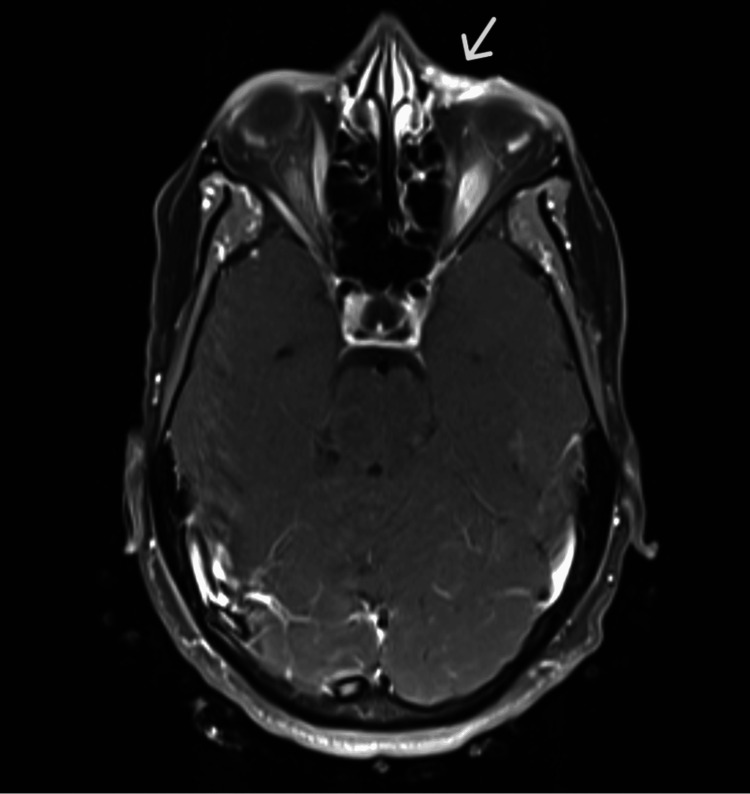
An MRI image showing enhancement in the left lower eyelid (with IV contrast)

**Figure 3:**
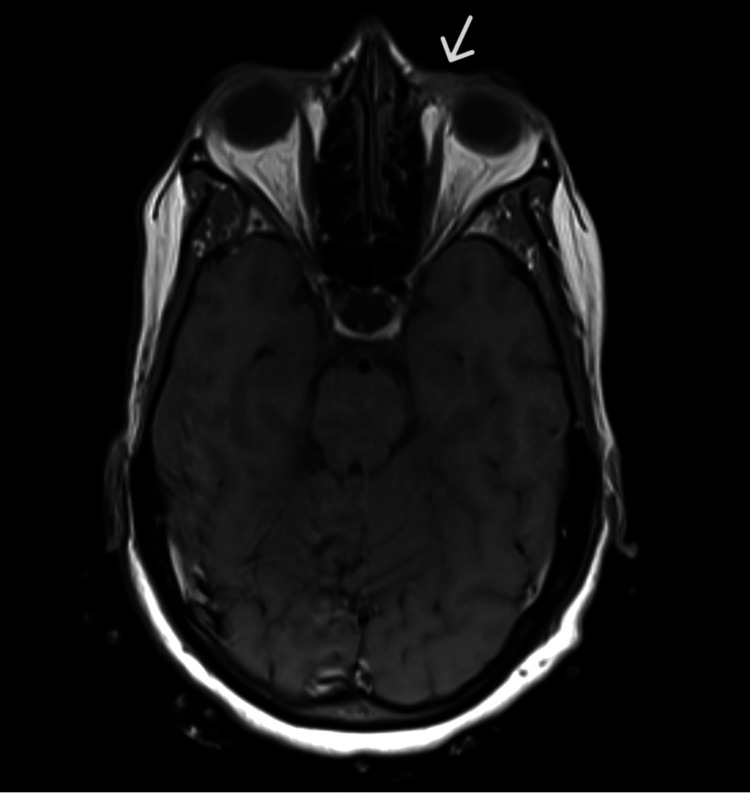
An MRI image showing a lesion in the left lower eyelid (without IV contrast)

An MRI of the face, orbit, and neck showed an enhancing soft tissue mass in the medial left lower eyelid region. No other significant abnormality was noted in the orbit (Figures 2-3).

The patient underwent excision of the lesion with lacrimal probing. Opthalmology felt that it was a difficult area for debulking and subsequent reconstruction, so they ruled out further surgical intervention, and she was referred for radiation. The patient subsequently completed radiotherapy using appositional 7 megaelectron volt (MeV) electrons (using skin collimation) and a 10 mm bolus; 40 Gy in 2 Gy fractions were delivered over four weeks. She is scheduled for follow-up in four months for re-evaluation, including serum electrophoresis and immunoglobulins.

## Discussion

We described a case of SEP in a 49-year-old female, diagnosed appropriately through an excisional biopsy and confirmed by the absence of monoclonal cells on a bone marrow biopsy and the absence of osseous uptake on a PET scan. The treatment was radiation therapy after an incomplete surgical excision at the lesion site. However, due to the location of the mass, this treatment was challenging as the eye shield required anesthetizing the left eye before each treatment. In addition, she developed some expected xerophthalmia from the procedure.

Plasma cell neoplasms arise as a result of the abnormal proliferation of mature B-lymphocytes, leading to the production of monoclonal immunoglobulins [6]. They can present in the absence of bone marrow or systemic symptoms and occur in the bones (solitary bone plasmacytoma) or without osseous involvement (solitary extramedullary plasmacytoma).

Only six confirmed cases of eyelid SEP have been reported in the literature (Ahamed et al., 2003; Olivieri et al., 2000; Seddon et al., 1982; Maheswari et al., 2009; Ria et al., 2006; Lim et al., 2013) [7-12]. The age at diagnosis in these cases ranged from 33 to 74 years, which falls within the age range suggested by Dores et al. in an observational study in 2009 [1]. Two cases presented with lower eyelid mass, while one was in the upper eyelid. All were treated with surgical excision. Interestingly, one patient received radiation after an incomplete excision, just like our patient.

Several risk factors have been noted to increase the likelihood of progression of solitary plasmacytoma of bone to multiple myeloma, including osseous tumors of more than 5 cm, lymph node involvement, and old age in patients [13]. However, no risk factors have been noted specifically for SEP.

Treatment options for extramedullary plasmacytoma include surgery, radiotherapy, or chemotherapy. Solitary extramedullary plasmacytomas are radiosensitive, with a response rate as high as ~93%-95% [13]. The optimal radiotherapy dose is at least 30 Gy, although most centers use doses between 40 and 50 Gy [14]. Radiotherapy is sometimes given after surgical excision, especially for patients with orbital plasmacytoma, which occasionally requires enucleation [14].

Radiotherapy has been recommended as standard therapy for extramedullary plasmacytomas; however, complete surgical excision provides similar results [14]. Alexiou et al. (1999) reported a lower rate of progression to MM in patients treated with surgery [5], but this may be related to size as smaller lesions are more amenable to surgical resection. Chemotherapy is only recommended in relapsed or refractory cases as an adjunctive measure. For patients who progress to MM, chemotherapy is the therapy of choice [5].

Ria et al. reported a case of SEP of the eyelid, which recurred with lymphadenopathy within two years of surgical excision [11], hence highlighting the importance of lifelong monitoring for the development of MM. The recurrence rates of SEPs after treatment have been reported to range from 6%-10% [15]. No data are yet available regarding the eyelid's SEP survival rate. Our patient is being followed by radiation and medical oncology and had no evidence of recurrence at the six-week follow-up.

## Conclusions

In conclusion, primary extramedullary plasmacytoma of the eyelid is rare. Clinicians should be aware of this tumor when patients present with an atypical recurrent eyelid mass like a progressively enlarging cystic lesion with positive illumination, which is not consistent with typical dacryocystocele. Systemic examination, relevant clinical investigations, and lifelong monitoring are required for these patients due to the strong association of extramedullary plasmacytoma with MM. Future prospective studies are needed to identify risk factors for the progression of SEP.
